# Pre-hospital Identification of a Giant Bladder Calculus through Screening Sonography: A Case Report

**DOI:** 10.2174/0115734056324600241114055235

**Published:** 2025-01-24

**Authors:** Sérgio Miravent, Carla Gomes, Paula Simãozinho, Bruna Vaz, Manuel Duarte Lobo, Rui Pedro de Almeida

**Affiliations:** 1 Basic Emergency Service of Vila Real de Santo Antonio, Algarve Local Health Unit Portugal; 2 Higher School of Health, Department of Radiology, University of Algarve, Faro, Portugal; 3 Faculty of Medicine and Biomedical SciencesUniversity of Algarve, Faro, Portugal; 4 Local Health Unit of Northeast, Polytechnic, University of Castelo Branco, Castelo Branco, Portugal; 5 Algarve Local Health Unit, Central ACeS Department; 6 Medical Imaging and Radiotherapy Department, Center for Studies and Development in Health (CES), University of Algarve, Faro, Portugal; 7 CHCR - Comprehensive Health Research Center; CICSNOVA - Interdisciplinary Centre of Social Sciences, University of Évora, Évora, Portugal

**Keywords:** Calculus, Cystolithotomy, Emergency, Renal, Screening, Ultrasound

## Abstract

**Introduction:**

Screening ultrasound proves to be remarkably beneficial in pre-hospital settings, particularly in geographically remote areas with technological constraints and no medical specialties. Urological pathology has a high frequency of occurrence in the emergency department and is part of the wide range of occurrences that can benefit from this ultrasound screening as a clinical guide for patients.

**Case Presentation:**

In this case, a patient experiencing lower abdominal pain and symptoms of renal colic sought assistance at a basic emergency service facility. Utilizing a renal screening ultrasound executed by a sonographer, the clinical team identified images indicative of a significant bladder calculus. Subsequently, the patient was referred to a referral hospital for a comprehensive evaluation by medical specialties.

**Conclusion:**

The images obtained in both health units exhibited congruence, indicating that the screening ultrasound, while not intended to replace the specialized orthodox ultrasound executed by a radiologist, served as a crucial tool for diagnostic presumption, providing consistency in clinical decision-making for referring patients. This capability allowed emergency physicians to promptly transfer a patient requiring urgent further investigation to a referral hospital with compelling and substantiated data. This shift in the approach to patient triage in a remote setting could enhance patient safety.

## 1 INTRODUCTION

Screening ultrasound is employed to address straightforward clinical queries and is performed globally by several healthcare professionals with different degrees of sonography formation and background [1-4]. Screening ultrasound, beyond its potential to save lives, significantly reduces diagnostic uncertainty, accelerates clinical decision-making, and shortens patient pathways, ultimately contributing to time and cost savings in healthcare services. Its integration into various emergency medical protocols, notably renal and urological pathology [5-8], is extensively documented in the scientific literature, emphasizing its role in emergency medicine. Point of Care Ultrasound (POCUS) applications for the bladder study in the emergency department are mainly used in bladder volume estimation, bladder mass, bladder outlet obstruction, hematuria, hydronephrosis, anuria, flank or pelvic pain, and confirming proper placement of Foley catheter [9, 10]. Nevertheless, apprehensions related to patient safety arising from the improper application of POCUS are rooted in the inadequate comprehension of the evidence base supporting this imaging modality. The inappropriate use of POCUS by less experienced medical personnel accentuates the crucial necessity for rigorous training and efficient response to prevent diagnostic mistakes and, in serious situations, avoid potentially fatal outcomes. This clinical report, divided into sections for clinical and imaging descriptions, discussion, and conclusion, highlights the crucial role of ultrasound in pre-hospital settings. It underlines how ultrasound guides patient care through imaging-based evidence, optimizing human and technological resources. By shortening diagnostic time and enhancing accuracy, ultrasound can help reduce the need for repeated emergency appliances.

## 2 CASE DESCRIPTION

A 49-year-old woman presented to a Basic Emergency Service (BES) for the seventh time in a two-year period, experiencing recurring symptoms consistent with renal colic and cystitis. In the Manchester triage, the patient was classified as orange (very urgent) with an abdominal pain score of 8 (0-10), accompanied by nausea and vomiting. She was afebrile, with normal blood pressure, oxygen saturation at 99% in atmospheric air, eupneic at rest, and showed no signs of respiratory distress. Abdominal palpation revealed pain on superficial and deep palpation in the hypogastrium, positive left renal Murphy’s sign [11, 12], and positive bowel sounds. The simple urine test (combur) indicated fetid-smelling urine with triple positive crosses for leukocytes and proteins and four crosses for hematuria.

**Fig. (1):**
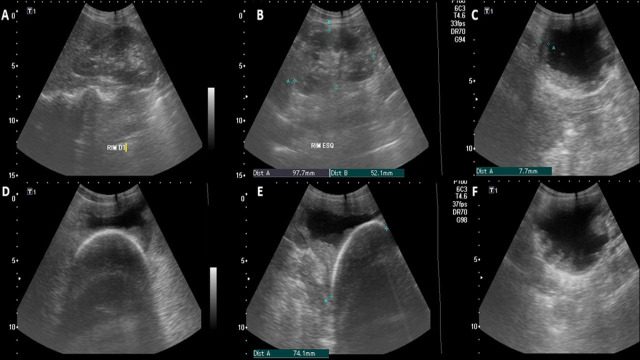
(**A**) Right kidney of normal appearance. (**B**) Left kidney with apparent increased medullary echogenicity in comparison to contralateral kidney. (**C**) An axial image of the bladder showing an increase in parietal thickness of approximately 8mm; on the live image, suspended echoes were evident. (**D**) An axial image of the bladder in a lower section showing a hyperechoic image measuring approximately 75mm axially. (**E**) Longitudinal image of the bladder revealing a hyperechogenic image measuring approximately 74mm in the longitudinal axis. (**F**) An axial image of the bladder showing an increase in parietal thickness and irregularity with different degrees of echogenicity.

Following anamnesis and physical examination, the emergency physician requested a focused renal screening ultrasound, partially depicted in Fig. (**1**). During the ultrasound examination, the patient experienced pain, notably exhibiting a positive sonographic Murphy's sign in the left flank [13]. The screening ultrasound revealed a bladder calculus measuring approximately 72.4 mm long and 60 mm wide, as depicted in Figure 1, specifically in images D and E, which show axial and longitudinal sections, respectively. In the central part of the images, a hyperechoic curvilinear structure can be visualized, corresponding to the upper surface of the calculus, projecting an intense posterior acoustic shadow [14] obscuring visualization beyond the initial reflective layer of the ultrasound.

The bladder wall appeared thickened and irregular, measuring approximately 7mm [15, 16]. The bladder's contents appeared impure, although a direct link to infection could not be established [17, 18]. The left kidney presented with a slightly more echogenic medullary segment compared with the same segment of the contralateral kidney, with no evidence of pyelocalyceal dilatation or free fluid in peritoneal recesses. Based on the clinical signs and ultrasound findings indicating a probable voluminous bladder stone [19, 20] and increased bladder wall thickness, a hypothesis of bladder inflammatory process was considered [21]. Supported by this data, the patient was referred to the referral hospital (RH) for additional imaging exams and a detailed renal function study.

While still in the BES, the patient received non-steroidal anti-inflammatory medication, opioid analgesic, antiemetic, and analgesic/antipyretic medications. Fig. (**1**) summarizes the main ultrasonographic images obtained at BES by a sonographer.

Upon arrival at the RH, the patient underwent comprehensive blood tests and abdominal and renal ultrasound in the Imaging Department. Blood tests showed normal parameters, except for a slight decrease in the erythrocyte series. The patient's erythrocyte count was 3.84 x10^12/L (4.60 - 5.20), hemoglobin 111 g/L (115 – 155), and hematocrit 0.33 L/L (0.35 - 0.45). Blood nitrogen and urea (BUN), creatinine, and C reactive protein (CRP) were normal. The partially transcribed ultrasound report by radiologists in Fig. (**2**) concluded, “bladder with non-pure contents (echoes in suspension) suggesting sediment and marked diffuse parietal thickening consistent with urinary infection. Calculus of approximately 76 mm inside the bladder”. Fig. (**2**) summarizes the main ultrasonographic images obtained at RH by a radiologist.

The patient continued to receive non-steroidal and opioid analgesics and remained hospitalized for 2 days under observation and control of cystitis. She left the RH with ambulatory instructions to follow up with the Urologic Department. Due to the pandemic context, the computed tomography (CT) execution and subsequent surgery to remove the stone were postponed.

Six months later, the patient underwent a CT for a complementary study, as summarized in Fig. (**3**), with two images of multiplanar reconstructions and a partially transcribed CT report. “In the left kidney, two oval images were identified, spontaneously hyperdense, probably related to cysts with hemorrhagic content/high protein content, measuring 16 mm and 7 mm. Perinephric spaces without significant changes. Bladder full, containing voluminous lithiasis formation measuring approximately 80 mm in the longest axis.” Fig. (**3**) presents a summary of the main computed tomography images obtained at RH.

The patient experienced two recurring episodes of cystitis before undergoing cystolithotomy for the removal of the calculus [22, 23]. Following the operation, the patient successfully recovered and has since been under routine follow-up in the urology department.

## 3 DISCUSSION

Bladder calculi of dimensions similar to the one in this case are considered giant and rare in international literature [24-26]. The ultrasound findings in the Basic Emergency Service (BES) were validated by the imaging department of the Referral Hospital (RH) through orthodox sonography and CT. The BES physician, using renal Point-of-Care Ultrasound (POCUS), successfully identified a giant calculus in the bladder and observed changes in wall thickness. Correlating these findings with the clinical presentation led to the hypothesis of probable cystitis. Typically, imaging examinations, such as ultrasound and CT scans, are reserved for situations where empirical treatment for cystitis or pyelonephritis proves ineffective. These imaging studies are crucial for identifying complications and evaluating structural or functional changes in the urinary system [27], as demonstrated in this clinical case.

Despite the high sensitivity and specificity values reported in the literature for renal POCUS when applied to urological pathologies by healthcare professionals [28, 29], it is essential to recognize that point-of-care screening ultrasound is not designed for definitive diagnoses [30]. A sizable proportion of patients with kidney issues may have a history of previous renal colic episodes [31]. Therefore, early identification of acute or chronic inflammatory conditions through renal POCUS and subsequent resolution holds significant value. This timely detection can effectively reduce the likelihood of cyclic recurrences, preventing hospital emergencies and thereby reducing associated healthcare costs while alleviating patients suffering from complications [32]. A large calculus is not typically the first clinical hypothesis considered when a patient presents with symptoms like those in this case. Therefore, given the unusual nature of its presentation, it is essential to emphasize the importance of incorporating such cases into sonographer training programs. This should include theoretical instruction, as well as practical exposure through images and videos of similar cases to enhance diagnostic accuracy.

## CONCLUSION

The decision to transfer the patient to the referral hospital was heavily reliant on the information gathered from the screening ultrasound. It is crucial to highlight that this incident occurred during a pandemic, requiring a well-substantiated clinical justification for patient referrals to specialized care. It is believed that screening ultrasound can play a pivotal role in early diagnosis, especially in situations where access to specialized care and advanced diagnostic resources is restricted due to the usual pressure on specialty departments in referral hospitals. Clinical teams where screening ultrasound is used have a greater potential to achieve better outcomes for patients and greater resource savings.

## Figures and Tables

**Fig. (2):**
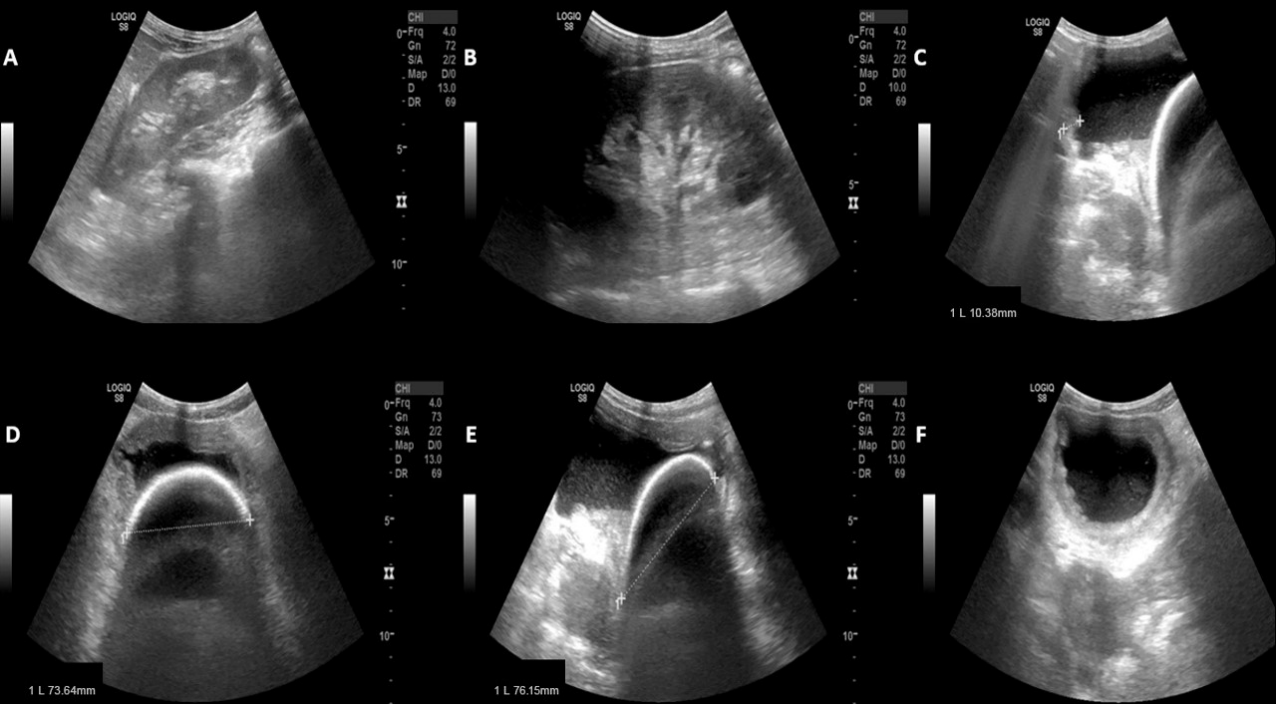
(**A** and **B**) Right and left kidneys with normal dimensions, regular contours, normal parenchymal thickness, and adequate parenchymal-sinus differentiation. (**C**) An axial image of the bladder with non-pure contents (echoes in suspension) is noteworthy, suggesting sediment and marked diffuse parietal thickening in accordance with the urinary infection. (**D**) An axial image and (**E**) a longitudinal image of a calculus with approximately 76mm longitudinal aspect and 73.6mm axial perspective inside the bladder. (**F**) An axial image of the bladder showing an increase in parietal thickness and bladder with non-pure contents.

**Fig. (3):**
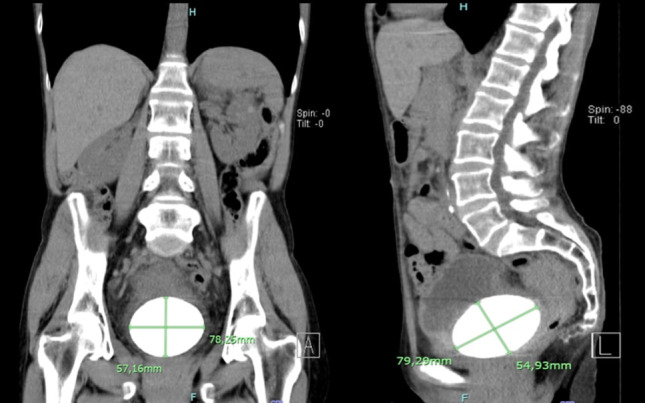
Coronal and sagittal multiplanar reconstruction highlighting the dimensions 57.26mm by 78.25mm and 79.29mm by 54,93mm, respectively, of the large stone as well as the most anterior and superior position of the bladder.

## Data Availability

The data sets used and/or analysed during this study are available from the corresponding author [S.M] upon request.

## LIST OF ABBREVIATIONS

POCUS: Point Of Care Ultrasound
BES: Basic Emergency Service
RH: Referral Hospital
BUN: blood nitrogen and urea
CRP: C reactive protein
CT: Computed Tomography

## STANDARDS OF REPORTING

CARE guidelines were followed.
